# Clinical, Laboratory, and Therapeutic Analyses of 21 Patients with Neonatal Thrombosis and Antiphospholipid Antibodies: A Literature Review

**DOI:** 10.1155/2014/672603

**Published:** 2014-07-17

**Authors:** Marcus Vinicius da Costa Peixoto, Jozélio Freire de Carvalho, Carlos Ewerton Maia Rodrigues

**Affiliations:** ^1^Universidade de Fortaleza (Unifor), 60811-905 Fortaleza, CE, Brazil; ^2^Aliança Medical Center, 41920-000 Salvador, BA, Brazil; ^3^Universidade Federal do Ceará (UFC), 60430-160 Fortaleza, CE, Brazil

## Abstract

*Objectives*. A review of the literature reports neonatal thrombosis and antiphospholipid antibodies cases through a retrospective study that focuses on the pathogenesis and main clinical and laboratory manifestations of this disease. *Methods*. The case reports were selected from PubMed. The keywords used to search were neonatal, antiphospholipid syndrome, thrombosis, and antiphospholipid antibodies. References that were published from 1987 to 2013 were reviewed. *Results*. Twenty-one cases of neonatal thrombosis and antiphospholipid antibodies were identified. Ten children were born preterm (before 37 weeks). Arterial involvement (17/21) was predominant, of which stroke (12/17) was the most prevalent clinical manifestation. Anti-cardiolipin antibodies were predominant (13/21) in the antiphospholipid antibody profiles. Treatments were based on the use of symptomatics such as antiepileptics (8/21), and 6/21 patients received heparin. There were 4 deaths (4/21); otherwise, the children recovered well, especially the neonates who suffered from strokes (9/12). *Conclusion*. Neonatal thrombosis and antiphospholipid antibodies are rare. The development of thrombotic manifestations in neonates seems not to be associated exclusively with the aPL, but their etiology may be linked to pre- and perinatal events. We noted good therapeutic responses, especially in stroke patients, who presented with favorable outcomes in 82% of the cases.

## 1. Introduction

Antiphospholipid syndrome (APS) is a multisystem autoimmune disease that is characterized by venous and arterial thrombosis, which may or may not be associated with pregnancy complications in the presence of antiphospholipid antibodies (aPL) [1, 2]. While APS in adults has been well characterized, only rarely have cases of thrombosis been reported in the neonates of mothers with APS or aPL. Thirty percent of children born to mothers who have aPL acquire these antibodies passively, and occurrences of thrombosis appear to be extremely rare [3]. Between 1987 and 2013, only 21 reports of neonatal thrombosis and aPL were published [1, 4–22]. In addition, in a large cohort, the onset of APS before age 15 occurred in 2.8% of the cases [23].

The current study aims to review the existing case reports in the literature with regard to neonatal thrombosis and aPL through a retrospective study that focuses on the pathogenesis and the main clinical and laboratory manifestations and outcomes in these patients.

## 2. Methods

Patients were considered as having neonatal thrombosis associated with the presence of aPL if the onset of thrombotic episode occurred before 28 days after birth due to the presence of aPL [3].

The case reports in the literature were selected from PubMed and case series were excluded because they do not present well-structured papers that could respond to our study questions. The keywords used were neonatal, antiphospholipid syndrome, thrombosis, and antiphospholipid antibodies. References that were available in English, Spanish, and French and published during the period from 1987 to 2013 were reviewed. The articles were reviewed for demographic characteristics (race and sex), clinical manifestations (symptom onset and evolution), obstetric conditions (delivery type and maternal history), treatment, and immunological markers. Laboratories tests used for determining aPL and anti-beta-2-glycoprotein I (anti*β*2GPI) were ELISA (Enzyme Immunoassay kit). Lupus anticoagulant (LAC) was detected using activated partial thromboplastin time and diluted Russel's viper venom time according to international guidelines [24]. Favorable outcome is defined such as a complete resolution of neurologic deficit. A total of 20 articles were found, 1 of which described 2 cases. Thus, a total of 21 neonatal thrombosis and aPL cases were found.

## 3. Results

Twenty-one cases of neonatal thrombosis and aPL were identified in 20 articles that were published as case reports during the period from 1987 to 2013.

The study reviewed 21 children, of whom 12 (57%) were males, 5 were females, and 4 were not specified. The average time of symptom onset occurred mainly within the first 48 hours (12/22). Cesarean section deliveries were reported in 11/20 cases due to preeclampsia (2), no progression of the delivery or fetal distress, and vaginal deliveries were reported in 7 cases. The gestational ages ranged from 27 to 41 weeks. Ten children were born preterm (before 37 weeks). Two children presented with sepsis and 2 with pneumonia. Two children required a central catheter.

Six mothers had only aPL without evidence of autoimmune disease, 7 had primary APS, 2 had secondary APS, and 6 were healthy. With regard to the aPL profiles in the neonates, 13 children had anti-cardiolipin antibodies, 6 had lupus anticoagulants, 4 had anti-*β*2GPI, 2 were double-positive, and 2 were triple-positive. Four children were included in a study of hereditary thrombophilia (Table 1).

Arterial involvement was present in 17 of the 21 cases. Stroke was the most prevalent clinical manifestation, occurring in 12 of the 17 cases, along with impairment of the middle cerebral artery (10/17). The aorta, mesenteric, and femoral arteries were involved in 2 cases and the renal artery in 1 case. Venous involvement was observed in 5 of the 21 children; in 3 of them, only the peripheral circulation was affected while, in the other 2, the central circulation was also involved. Convulsions were observed in 9 of the 21 patients; these were usually secondary to stroke, and thus the development of epilepsy was uncommon and reported in only 1 patient. One patient developed catastrophic APS, another presented with Sneddon's syndrome, and a third presented with thrombocytopenia.

The treatments were mainly based on the use of symptomatics such as antiepileptic drugs (8/21). One patient was treated with immunoglobulin for thrombocytopenia, 2 patients were subjected to thrombolysis (thrombosis of the femoral artery and aorta), and 6 patients received heparin. Fourteen (66.6%) of the 21 children had favorable outcomes, 2 had partial improvements (1 persisted with monoparesis in the left arm and another developed epilepsy), and 4 (19%) died (renal vein thrombosis, mesenteric artery thrombosis, aortic thrombosis, and subclavian and jugular vein thrombosis). Of the 11 children who had strokes, 9 had favorable outcomes. The mean time of follow-up was 1 at 5 years (Table 1).

## 4. Discussion

### 4.1. Pathogenesis

It has been suggested that pathogenic aPL could be absorbed at the placental level. Since placental passage is limited, this may explain the relative low frequency of clinical events in neonates [25]. Despite the strong association between thrombosis and the presence of aPL in neonatal APS, according to the cases published in the literature, thrombosis is associated with other risk factors such as prematurity, respiratory infections, sepsis, asphyxia, dehydration, central catheters, and parenteral nutrition. Furthermore, the low levels of antithrombin III, protein C, and protein S as well as mutations in the genes for factors V and II are predisposing factors [25, 26].

Autoimmune diseases are very rare in newborns due to their immature immune systems, which will become fully developed by the age of 3 [27]. This is why the majority of autoimmune diseases in newborns occur in response to the transplacental passage of diseases from mothers to newborns. However, there are numerous cases of pregnancies in which aPL were passed transplacentally but there were no clinical manifestations of APS. This further corroborates the idea that other factors in addition to the presence of aPL are needed to induce thrombotic phenomena in neonates and these factors may function as a trigger for the occurrence of thrombosis, like a “second hit model” [1–5, 10]. Many women of reproductive age might have several types of autoantibodies; however, these women remain asymptomatic, and the antibodies are only detected when their offspring suffer from the effects of transplacental passage [4]. Furthermore, it is possible that the neonates of mothers with negative aPL produce aPL, which suggests the de novo production of these antibodies [1, 2]. Moreover, other associated risk factors seem to be associated besides the presence of autoantibodies. Such factors might be associated with pre- and perinatal events or prothrombotic gene mutations that lead to coagulation system dysfunction [1, 2, 6, 11]. Accordingly, a 5-year prospective follow-up study evaluated the immunological status and evolution of 134 children who were born to mothers with APS and found no instances of thrombosis or systemic lupus erythematosus despite the presence of lupus anticoagulant (4%), IgG anti-cardiolipin antibodies (16%), and IgG and IgM anti-*β*2GPI 15% and 3%, respectively [28].

Among the prenatal risk factors, gestational diabetes, preeclampsia, intrauterine growth restriction, maternal APS, and systemic lupus erythematosus (SLE) are predisposing factors to thrombosis in neonates [29]. Of the 21 cases reported, 9 mothers had APS and among them, 1 developed SLE after pregnancy and another was diagnosed with APS only because of the clinical manifestations observed in her newborn [1, 2]. Despite having no history of thrombotic events, 6 other case reports demonstrated positivity of at least 1 antiphospholipid antibody; these 6 mothers were healthy, which suggests the de novo production of antiphospholipid antibodies by the neonates [7, 9–11, 13, 16–22]. Within the case reports, anti-cardiolipin antibody appeared to be the most common disease-associated antibody, and its presence was detected in 62% of the newborns.

Prematurity, asphyxia, respiratory infections, sepsis, central catheter, and parenteral nutrition seem to be the most common perinatal risk factors [29, 30]. The detailed analysis of the 21 cases demonstrated the presence of perinatal risk factors in addition to the aPL. In this review, 57% of the children had some of these risk factors, and prematurity was the most frequently reported factor (48%).

Another risk factor that appeared to be associated with the thrombotic events in neonates is congenital thrombotic gene mutation. Deficiencies in proteins C and S and mutations in the coagulation factors appear to be associated with thrombotic events in newborns, thus reflecting an imbalance between the coagulants and anticoagulant activity [16–18, 22].

Gene mutations were observed in 4 cases [16–18, 22]. Hereditary prothrombotic conditions were routinely investigated in previous studies of neonatal APS, particularly mutations in the methylenetetrahydrofolate reductase C677T and prothrombin G20210A genes that could explain the low prevalence of these prothrombotic factors in most reported cases [16]. According to these results, a multicenter study of children with APS observed that hereditary thrombophilia was more common in children who experienced a single episode of thrombosis (8 (53.3%) of 15 patients) than in those who experienced recurrent episodes (2 (28.6%) of 7 patients) [29]. Simchen et al. [31] investigated the roles of maternal and neonatal thrombophilia in a cohort of 47 newborns. Thirty children had evidence of thrombophilia. Factor V Leiden, protein C deficiency, and aPL all increased the prevalence of perinatal stroke and were important to stroke pathogenesis in this age group. This study suggests that maternal and neonatal thrombophilia, especially the Factor V Leiden and aPL, could be important in perinatal ischemic stroke pathogenesis [31].

### 4.2. Clinical Manifestations

#### 4.2.1. Deep Vein Thrombosis

Unlike adults, in whom deep venous thrombosis was found in 39% of the cases [32], the reported prevalence of deep vein thrombosis (DVT) is lower in neonates (24% of the cases). Only 5 cases of venous thrombosis were reported in the studies included in this review [1, 7–9, 19]. We observed 1 case of a neonate [8] who developed thrombosis in the aorta, left renal artery, middle cerebral artery, and superior sagittal sinus. De Carolis et al. [20] reported the case of a newborn who experienced severe respiratory distress caused by pneumonia soon after birth. A routine ultrasound identified a hyperechoic area on the right side of the Sylvian fissure. Despite the absence of clinical symptoms, magnetic resonance imaging showed thrombosis in the superior sagittal sinus.

In a study that evaluated the risk of thrombosis in pediatric patients, 28 children with APS at an average age of 13 years were analyzed; venous thrombosis, stroke, and thrombocytopenia were the most commonly observed manifestations [30]. Another study assessed the outcomes of renal vein thrombosis in 28 newborns and observed that, of 11 neonates evaluated for congenital thrombophilia, 1 had the factor V Leiden mutation; however no neonate was evaluated for aPL. One mother had antiphospholipid syndrome. Maternal diabetes was present in 5 mothers (18%) and 25 (89%) and 3 (11%) of the neonates had unilateral and bilateral involvement, respectively [33].

#### 4.2.2. Stroke

Perinatal stroke occurs in 1 of every 4000–5000 live births [31]. Despite the low incidence of clinical APS manifestations in neonates, the brain, the kidneys, and the heart are the most affected organs [2, 3]. In this review, the brain was the site most affected by the thrombotic events, and the cerebral artery was the most commonly affected site in the brain (59% of the cases), a higher prevalence than what has been reported in the literature (50% of cases) [24]. Another interesting finding was that 82% of the children who presented with stroke (11/21) progressed favorably without any motor deficit or seizures.

Newborns from mothers with aPL had an increased risk of stroke [30]. Eighteen percent of the mothers were positive for aPL, compared to 4.7% of the controls; however, this association had already been reported by another study [33]. Accordingly, perinatal stroke has received attention as an important cause of cerebral palsy and other neurological disabilities, including epilepsy and cognitive impairment [34–36]. However, in our review, we found only 2 cases of neurological disabilities. In one case, the patient developed epilepsy, and in the other case, the patient presented with motor deficits [14, 22], reinforcing the idea of a favorable outcome for newborns with stroke. These results might reflect a better prognosis for patients who receive early and effective treatment, rather than a peculiarity of this patient group or a bias in the reporting of cases considered to be relevant.

Although stroke and TIA correspond to 50% of the arterial thrombosis cases [25], these are not the only conditions. This fact was demonstrated by a case report of a 10-day-old newborn who presented with a bluish discoloration in the lower limbs, the absence of a popliteal pulse and pedal pulse, and a prolonged capillary refill time. A left femoral artery thrombosis was revealed and found to be associated with* Klebsiella* sepsis [19].

#### 4.2.3. Convulsive Seizures

Convulsive seizures usually have 2 peaks of incidence. One occurs in the first year of life, during which pre-, peri-, and postnatal processes facilitate central nervous system aggression. The other peak occurs in the seventh decade of life, during which there is a higher incidence of degenerative neurological diseases [37]. The nervous system aggression is an abnormal electrophysiologic phenomenon that occurs in the brain and results in abnormal discharge of electrical neuronal activity [37].

During the neonatal period, seizures are mainly attributed to neonatal asphyxia, metabolic disorders, or infections [38]. Thromboembolism is a relatively rare cause of seizures in newborns and is often secondary to intravascular catheters [39]; however, it can be associated with many conditions such as thrombophilia. Convulsive seizures often result from cerebral ischemia. We observed that 43% of the patients in this study had seizures as a major clinical manifestation and that these were usually secondary to cerebral artery infarcts. Thus, epilepsy development was unusual [10, 11, 14–17, 20, 21]. Alshekaili et al. [17] described the case of a 5-day-old newborn who had a reduction in spontaneous right limb movements. Many nonfebrile seizures occurred in the sixth month of life. Computed tomography revealed an area of middle cerebral artery infarction. Laboratory tests showed the presence of anti-cardiolipin and anti-*β*2GPI antibodies in neonatal serum but they were absent from cord blood and maternal serum. Additionally, this neonate also carried one prothrombotic allele of factor V (Leiden allele), which may have contributed to the risk of thromboembolic disease and the serological analysis represents unequivocal evidence of de novo neonatal primary APS. Likewise, a 13-hour-old girl had convulsive seizures that were associated with ischemia of the middle cerebral artery and mutations in prothrombotic genes [15].

#### 4.2.4. Sneddon's Syndrome

This syndrome is characterized by 3 main manifestations: ischemic stroke, livedo reticularis, and antiphospholipid antibody positivity [25]. In the current review, we found only 1 case that was suggestive of Sneddon's syndrome [17].

#### 4.2.5. Thrombocytopenia

Thrombocytopenia is a manifestation present in nearly 30% of APS cases [23]. We observed 1 case in which thrombocytopenia was evident. Soares Rolim et al. [1] reported a case of a 1-day-old newborn who exhibited respiratory distress in response to a respiratory infection. A week later, the newborn presented with thrombocytopenia and thrombotic events. In addition to having a mother with primary APS, the child had a high IgM anti-cardiolipin titer, which led the authors to conclude that this newborn produced aPL.

#### 4.2.6. Catastrophic Antiphospholipid Syndrome

Recently, the CAPS Registry studied catastrophic events in children and observed that 10.3% (45/446) patients were before 18 years of age. Overall, 32 (71.1%) patients were female and the mean age was 11.5 ± 4.6 years (range, 3 months–18 years). A total of 31 (68.9%) patients suffered from primary APS, 13 (28.9%) from systemic lupus erythematosus (SLE), and one from a lupus-like disease (2.2%). No one had neonatal CAPS [40].

We observed only 1 case report of catastrophic APS in which the newborn was triply positive for aPL associated with prothrombin and plasminogen activator inhibitor gene mutations that might have amplified the risk of thrombosis in this patient. Despite the triple positivity associated with prothrombotic gene mutations and the most serious manifestations of APS, the treatment was early and effective. Case of [17] reports a 17-day-old premature newborn who was admitted to emergency care with irritability, abdominal distension, vomiting, bloody stools, and signs of shock. A laparotomy was performed after stabilizing the patient, and necrotizing enteritis (transmural infarction) was found to be complicated by heart and kidney failures as the second hit, so it was part of the CAPS. Thirty months later, the child experienced ischemic stroke-induced tonic-clonic seizures.

### 4.3. Treatment

The lack of specific guidelines for neonates with thrombosis associated with aPL may be explained due to immaturity of the fibrinolytic system, suggesting that the patients should be followed up past puberty for validation of risk [41]. Neonates have a number of differences in hemostasis and fibrinolysis compared with adults that affect the incidence, treatment, and long-term outcome of thrombosis [41, 42].

Treatment for neonatal thrombosis and antiphospholipid antibodies comprises anticoagulant therapies such as aspirin, heparin, and warfarin [25]. A low prevalence for anticoagulant use was very common in the studies. Due to ischemic stroke, there is a consensus to avoid secondary prevention with antithrombotic agents in the majority of neonates. The rate of stroke recurrence was almost 0 in the majority of the studies [30, 31, 43]. In a large cohort of 215 children with neonatal stroke, only 7 had recurrent thrombotic events after a mean 3.5-year follow-up period [44]. Three of the children had thrombosis-related issues (congenital heart disease and congenital moyamoya disease) and 5 of them presented with a prothrombotic state alone or in combination with lipoprotein levels >30 mg/L and protein C deficiencies. Interestingly, none of the children with antiphospholipid antibodies had recurrences as reported in another study [30].

For neonatal thrombosis and antiphospholipid antibodies APS, in addition to the anticoagulants, treatments are based on the comorbidities present in the newborns; particularly, infections are treated with antibiotics and clinical manifestations such as seizures and respiratory distress are treated with antiepileptics (e.g., phenobarbital) and support therapy (e.g., mechanical ventilation), respectively. Other treatment modalities were offered; for example, 1 patient received immunoglobulin due to thrombocytopenia, [1] and another patient underwent thrombolysis with urokinase due to a thrombosis of the left femoral artery [19].

## 5. Limitations of the Study

There are no validated criteria for neonatal APS. The publications were heterogeneous with regard to the year of publication and advances in knowledge of the syndrome, such as the discovery of new antibodies and a better understanding of disease pathogenesis and the inclusion of new therapies.

## 6. Conclusion

Stroke was the most frequent clinical manifestation associated with antiphospholipid antibodies in neonates, and children who suffer strokes seem to develop favorably. Preterm birth was an additional perinatal risk factor for thrombotic manifestations.

From the findings in the current study, we conclude that neonatal thrombosis and antiphospholipid antibodies are a rare event, even in mothers who have antiphospholipid antibodies. The development of thrombotic manifestations in neonates seems not to be associated exclusively to the aPL, but their etiology may be linked to pre- and perinatal events or coagulation factor gene mutations.

## Figures and Tables

**Table 1:** Clinical, laboratory, and therapeutic analyses of 21 patients with neonatal thrombosis and antiphospholipid antibodies.

Case	Reference	Sex	Delivery and newborn age	Clinical manifestation	Maternal history	Neonatal antibodies	Treatment	Evolution
1	Finazzi et al. 1987 [4]	F	Preeclampsia cesarean delivery/20 days	Cyanosis in the left foot. US Doppler: left femoral artery thrombosis	Primary APS	Lupus anticoagulant	NR	Death on day 30 due to extensive aortic thrombus (autopsy)

2	Sheridan-Pereira et al. 1988 [5]	F	Cesarean delivery/at birth	DIC and MV. Absence of distal pulses and cyanosis. Thrombosis of the aorta at the origin of the superior mesenteric artery 32 hours after birth. Central catheter used	Maternal APS	Lupus anticoagulant	Plasmapheresis and heparin	The peripheral pulses were normal on day 13

3	Silver et al. 1992 [6]	NR	Delivery NR/at birth	Right spastic hemiparesis. MRI: middle cerebral artery infarction	Mother with multiple sclerosis and primary APS	NR	NR	Improvement of the hemiparesis at 6 months

4	Contractor et al. 1992 [7]	NR	Cesarean delivery/1 day	Left abdominal mass and hematuria. Renal vein and inferior vena cava thrombosis	Mother with aPL	IgG anti-cardiolipin	NR	Clinical improvement at day 7. The antiphospholipid profile disappeared at 4 months postpartum

5	Tabbutt et al. 1994 [8]	M	Cesarean delivery/3 days	Aortic, left renal artery, middle cerebral and superior sagittal sinus thrombosis, sepsis	Primary APS	Prolonged APTT, aPL negative	Heparin and ATB	Favorable at 2 months

6	Hage et al. 1994 [9]	M	Cesarean delivery/at birth	Hydrops fetalis with renal vein thrombosis	Healthy mother	Lupus anticoagulant	Mother was treated with tocolytic medications	Unfavorable. Fetal death

7	Teyssier et al. 1995 [10]	M	Normal vaginal delivery/3 days	Convulsion due to cerebrovascular ischemia and massive bilateral adrenal hemorrhage	Mother with aPL	Anti-cardiolipin positive	Phenobarbital	Favorable. Normal EEG at 7 months after birth

8	de Klerk et al. 1997 [11]	M	Normal vaginal delivery/at birth	Seizures, blinking repeatedly, spasms on the right side of the head, hand, and foot (no longer than 3 minutes). Ischemic stroke at the level of the left middle cerebral artery	Mother with aPL	Lupus anticoagulant	Phenobarbital	Favorable after 1 year

9	Navarro et al. 1997 [12]	NR	Cesarean delivery/3 days	Respiratory distress syndrome, pneumoperitoneum and abdominal livedo (mesenteric thrombosis)	Primary APS	IgG anti-cardiolipin	NR	Death on day 11 after laparotomy

10	Akanli et al. 1998 [13]	NR	Cesarean delivery/6 hours	Apnea, cyanosis, and moderate hypotonia. Mechanical ventilation. Progressed with stroke in the left middle cerebral artery area	Mother with aPL	IgG anti-cardiolipin	NR	Seizures until 3 years of age

11	Chow and Mellor 2000 [14]	F	Cesarean delivery/48 hours	Focal seizure in the left hemibody. CT: cerebral ischemia	Primary APS	IgG anti-cardiolipin	Phenobarbital and phenytoin	Favorable. Normal neurological examination 5 months after birth

12	Chow and Mellor 2000 [14]	M	Delivery NR/at birth	Right hemiplegia; later developed epilepsy. CT: ischemia at the level of the left middle cerebral artery	Primary APS	NR	NR	Unfavorable. Epilepsy

13	Tuohy and Harrison 2005 [15]	M	Preeclampsia, cesarean delivery/5 days	Anuria, paleness, and absence of pulse in the lower extremities. Aortic thrombosis	Primary APS	IgG anti-cardiolipin	rTPA	Death from renal failure on day 10

14	Soares Rolim et al. 2006 [1]	M	Delivery NR/20 hours	Thrombocytopenia, livedo reticularis; pericardial effusion; subclavian vein and external jugular thrombosis concomitantly with severe respiratory tract/central catheter infection	Secondary APS	IgG and IgM anti-cardiolipin	Immunoglobulin IV heparin and antibiotic treatment	Death

15	Paro-Panjan et al. 2007 [16]	F	Normal vaginal delivery/13 hours	Seizures; ischemic stroke. MRI: occlusion of the left middle cerebral artery	Mother with aPL	IgG anti-cardiolipin. Mutation of the prothrombin gene polymorphism G20210A and gene polymorphism C677T	Phenobarbital	No neurological deficits after 1 year of follow-up

16	Alshekaili et al. 2010 [17]	M	Normal vaginal delivery/5 days	Reduced spontaneous movement of the right limbs; ischemic stroke of the middle cerebral artery. Epilepsy. Late livedo reticularis (after 4 years)	Healthy mother	IgG anti-cardiolipin, IgG anti B2GPI. Factor V Leiden (G20210A allele)	Antiepileptic and anticoagulant	Favorable

17	Cabral et al. 2011 [18]	M	Normal vaginal delivery/17 days	Irritability, abdominal distension, vomiting, bloody stools with signs of shock, necrotizing enteritis-exploratory laparotomy. ICC: after 13 months, started to exhibit tonic-clonic seizures and right hemiparesis. Stroke in the frontal, parietal, and right temporal lobes	Healthy mother	Lupus anticoagulant, IgG/IgM anti-cardiolipin, IgG and IgM anti-B2GPI. PT polymorphism 20210G>A and PAI-1 polymorphism 675G>A	Low molecular weight heparin, acetyl salicylic acid, and phenobarbital	Favorable after 5 years of follow-up

18	Bhat et al. 2011 [19]	M	Cesarean delivery/3 days	Sepsis. Bluish discoloration in the left lower limb (thrombosis of the left femoral artery); absence of popliteal pulse and prolonged capillary refill time	Mother with aPL	Lupus anticoagulant	Thrombolysis, urokinase/heparin, and antibiotic	Favorable

19	De Carolis et al. 2012 [20]	M	Normal vaginal delivery/1 day	Severe respiratory distress due to pneumonia. Thrombosis in the superior sagittal sinus	Healthy mother	IgG anti-cardiolipin	NR	Normal neurological development after 1 year

20	Merlin et al. 2012 [21]	M	Normal vaginal delivery/3 days	Right clonic seizures. MRI showed left middle cerebral artery infarction	Healthy mother	IgG anti-cardiolipin and IgG B2GPI	100 mg of valproate and aspirin every other day	Normal development and neurological examination after 1 year

21	Sousa et al. 2012 [22]	F	Cesarean delivery/8 hours	Focal and multifocal seizures and left hemiparesis. MRI: ischemic stroke	Healthy mother	IgG anti-cardiolipin, IgG B2GPI, and lupus anticoagulant positives (de novo synthesis). 1298C/C MTHFR and PAI-1 844A/A and 675 4G/4G gene polymorphisms	Anticonvulsants and aspirin	L side body strength improved and monoparesis in the left upper limb persisted after 1 year. Seizures improved at 3 months of age

APS: antiphospholipid syndrome. DIC: disseminated intravascular coagulation. MV: mechanical ventilation. rt-PA: recombinant tissue plasminogen activator. aPL: antiphospholipid antibodies. MRI: magnetic resonance imaging. IV: intravenous. B2GPI: B2-glycoprotein I. PT 20210G>A: heterozygosity for the prothrombin gene. PAI-1 675G>A: homozygosity for the plasminogen activator inhibitor-1 gene. 1298C/C MTHFR: homozygous mutation of the methyltetrahydrofolate reductase gene. PAI-1 844A/A and 675 4G/4G: double heterozygosity for the inhibitor plasminogen activator. C677T: heterozygosity for the methylenetetrahydrofolate reductase gene polymorphism. NR: not reported.
